# Effect of green tea on the gastrointestinal absorption of amoxicillin in rats

**DOI:** 10.1186/s40360-019-0332-8

**Published:** 2019-08-30

**Authors:** Tivadar Kiss, Zoltán Timár, Andrea Szabó, Anita Lukács, Viktória Velky, Gábor Oszlánczi, Edina Horváth, István Takács, István Zupkó, Dezső Csupor

**Affiliations:** 1 Department of Pharmacognosy, Faculty of Pharmacy, University of Szeged, Eötvös utca 6, Szeged, H-6720 Hungary; 2 Interdisciplinary Centre for Natural Products, University of Szeged, Eötvös utca 6, Szeged, H-6720 Hungary; 3 SOLVO Biotechnology, Közép Fasor 52, Szeged, H-6726 Hungary; 4 Department of Public Health, Faculty of Medicine, University of Szeged, Dóm tér 10, Szeged, H-6720 Hungary; 5 Department of Pharmacodynamics and Biopharmacy, University of Szeged, Eötvös utca 6, Szeged, H-6720 Hungary

**Keywords:** Amoxicillin; green tea, *Camellia sinensis*, Pharmacokinetics, LC-MS/MS, Interaction

## Abstract

**Background:**

The investigation of food-drug and plant-drug interactions has become increasingly important. In case of antibiotics, it is essential to achieve and maintain a plasma concentration sufficient for the antimicrobial action. Although, on theoretical basis, the interaction of polyphenols and antibiotics may be hypothesized, experimental data are lacking to assess its clinical relevance. The aim of our study was to assess the interaction between one of the most widely used antibiotics, amoxicillin, and green tea, the most frequently consumed drink with high polyphenol content.

**Methods:**

The effects of green tea on the plasma level of amoxicillin was studied in an in vivo experiment in rats. The plasma level of amoxicillin was monitored by LC-MS/MS for 240 min after oral administration. The polyphenol content of green tea was determined by the Folin-Ciocalteu method.

**Results:**

The peak plasma concentration of amoxicillin significantly decreased upon its co-administration with green tea, although the AUC_0–240_ of the antibiotic did not decrease significantly in the group treated with amoxicillin suspended in green tea.

**Conclusions:**

Our results suggest a potentially relevant interaction between green tea and amoxicillin, worth being further studied in humans.

## Background

Optimal dosing and timing, as well as the route of administration and justified use essentially influence the outcome of antibiotic therapy. The excessive use of antibiotics, including overuse and inappropriate application (including using suboptimal doses) of antimicrobial agents fosters bacterial resistance, recognized as one of the most urgent global public health threats, and being the main reason for nosocomial infections [1]. Therefore, the World Health Organization (WHO) makes continuous efforts to raise the awareness of healthcare professionals and patients about proper antibiotic use.

Some aspects of antibiotic interactions have been extensively studied. It is well known that the co-administration of several antibiotics with milk products should be avoided as bivalent ions, i.e. calcium and magnesium in milk form complexes with these pharmacons, thereby decreasing their absorption [2]. Although some food and herb interactions with antibiotics have also been described, the number of studies focusing on this issue is limited. For the majority of medicinal herbs it is still undiscovered whether they alter the pharmacokinetics of antibiotics.

However, there are reasonable theoretical considerations that suggest the risk of potential interactions. Some components of medicinal plants may interfere with the bioavailability of antibiotics by decreasing their absorption. The two most important family of compounds that may potentially affect the pharmacokinetics of antibiotics include fibres and polyphenols, which most frequently occur in commonly used plants. These compounds may affect the absorption of different drugs via chemical or physical interactions. The effect of fibres (chemically polysaccharides) is non-specific, hence these compounds affect the bioavailability of various medicines, such as digoxin, lovastatin, metformin and penicillins [3]. Polyphenols are known to react chemically with amine-containing molecules resulting in precipitation which may lead to decreased bioavailability [4]. Although several drug molecules contain amine functionalities, this potential interaction has not been studied in detail. This possible interaction may be avoided by consuming polyphenol-rich food (e.g. fruits) separately from medicines, however, the risk of taking medicines with drinks containing polyphenols is neglected. The most popular drink with a remarkably high polyphenol content is tea, especially green tea. Tea is the most widely consumed beverage aside from water, and its consumption is growing worldwide [5]. Although, globally, black tea is Although, globally, the most frequently consumed the most frequently, even in countries where it is the top type of tea (e.g. Morocco, Turkey, Ireland and the United Kingdom), green tea consumption shows an increasing popularity due to a growing consumer interest toward healthier beverages [6]. Green tea is one of the most popular beverages in Japan, China, as well as in some countries in North Africa and the Middle East [7]. Generally, 40–70% of the Japanese population consumesconsume green tea regularly (at least 1 cup/day) [8, 9].In Europe is consumed as the top In Europe, green tea is the most type of tea in Belgium, Denmark and France [10]. The health benefits of green teaThe health benefit of green tea has been extensively studied: it has proved antioxidant, anti-inflammatory, anticarcinogenic, and antimicrobial effects [11, 12]. These health promoting effects are mainly associated with its polyphenol content, that may constitute up to 30% of the dry leaf weigh [7].

Based on its highly frequenthigh consumption, green tea is very likely to be consumed with medicines, including antibiotics. In a recent review [13], the interaction between green tea and clinically prescribed medications was summarized as follows: the plasma concentration of some drugs (e.g. diltiazem, clozapine, tamoxifen) changed significantly when they were administered concurrently with green tea/EGCG to male Sprague-Dawley rats. Nevertheless, the interaction between green tea and antibiotics has been poorly investigated. The majority of the available studies have been carried out in vitro, primarily to explore the potential synergistic pharmacodynamic interactions [14, 15]. Egyptian scientists found that even diluted green tea enhanced the bactericidal activity of several antibiotics against 28 microorganisms in vitro, and moreover, it increased the susceptibility of drug-resistant bacteria [16]. Noormandi et al. thoroughly summarized the synergistic effect of green tea on antibiotic treatment of urinary tract infections, however, they emphasized that despite the promising results of in vitro studies, human and animal studies are needed [17]. Based on the potential antagonistic (inhibition of absorption) and agonistic (increase of antibacterial activity) interactions, the overall effect of the concomitant consumption of green tea and antibiotics seems to be contradictorycontradictory.

Amoxicillin is one of the most commonly used antibiotics in primary care, prescribed either alone or in combination with clavulanic acid, with a remarkable dominance in inpatient therapy [18, 19]. It is reported that high-fiber diet reduces the bioavailability of amoxicillin [20–22], while regular type food intake [21, 23, 24] does not affect it. Interestingly, while most antibiotics interact with calcium, and thus the concomitant consumption of milk and antibiotics is not recommended, this phenomenon was not observed for amoxicillin [25, 26]. However, the amoxicillin molecule contains amine functionalities, thus it might be subject to interaction with polyphenols.

The interaction between green tea extract and amoxicillin was studied in vitro only. In one study synergistic interactions between ampicillin, a β-lactam antibiotic alike amoxicillin, and green tea against *Staphylococcus aureus* were described [15].. Also, in an in vitro study, *Passat* found that the water extract of green tea leaves has synergistic effect on some antibiotics including amoxicillin, while it has and antagonistic effect on other antibiotics used against urinary tract *E. coli* isolates [27].

As in vivo or toxicokinetic studies are still missing in the literature, thet aim of our research was to study this potentially relevant interaction, i.e. the effect of green tea on the absorption of amoxicillin, in an in vivo experiment on rats, as a preliminary step for a suggested human study. To the best of our knowledge, our work is the first in vivo toxicokinetic investigation of this issue in vivo.

## Methods

### Experimental substances

Amoxicillin trihydrate (product number: 31586) and potassium clavulanate (product number: 33454) were purchased from Sigma-Aldrich. Green tea (China-Chun Mee) was obtained from Hungary (Latin Negyed Ltd., Hungary). All the solvents were of LC-MS grade (VWR, Hungary).

### Determination of polyphenol content

Total polyphenol content, determined as pyrogallol equivalent, was measured according to the method described in the European Pharmacopoeia [28]. In a round-bottomed flask 0.5 g (m_1_) powdered green tea was extracted with 150 ml of water in a water-bath for 40 min. After cooling under running water, it was transferred to a 250 ml volumetric flask and was diluted to 250.0 ml with water. The extract was filtered through a filter paper of 125 mm in diameter. The first 50 ml of the filtrate was discarded. 5.0 ml of filtrate was diluted to 25.0 ml with water. 2.0 ml of this diluted extract was transferred to a 10 ml volumetric flask, 1.0 ml of two-fold dilution of Folin-Ciocalteu reagent was added, and the mixture was diluted to 10.0 ml with a 290 g/l solution of sodium carbonate. After 30 min absorbance at 760 nm (A_1_) was measured. For a standard solution 0.0500 g (m_2_) of pyrogallol was dissolved in water immediately before use, and diluted to 100.0 ml using the same solvent. 5.0 ml of this solution was diluted to 100.0 ml with water. 2.0 ml of this diluted pyrogallol solution was transferred to a 10 ml volumetric flask, 1.0 ml of two-fold dilution of Folin-Ciocalteu reagent was added, and the mixture was diluted to 10.0 ml with a 290 g/l solution of sodium carbonate. After 30 min absorbance at 760 nm (A_2_) was measured. For the calculation of total polyphenols as pyrogallol equivalent (PGE, %), the next formula was used:

$$ \frac{62.5\cdot \kern0.5em {A}_1\cdot \kern0.5em {m}_2}{A_2\cdot {m}_1} $$

### Test animals and animal care

24 male SPF Wistar rats, weighing 276–300 g at the beginning of the study, were used in the experiment (purchased from Toxi-Coop Zrt., Hungary). After 5 days of acclimatization the rats were divided into two well-balanced groups according to body weight (antibiotic only group (AM) and antibiotic plus green tea group (AMG), 12 animals/group). The number of animals per group was calculated to maximize the number of blood sampling occasions while minimizing the number of animals used for blood sampling per occasion. As environmental enrichment, unbleached, clean paper tubes were provided for the rats. Three rats per cage were placed on dust-free wood shavings as bedding material. Animals were housed under standard climatic conditions (22–24 °C, 30–70% relative humidity, 12 h light/dark cycle with light starting at 6:00 a.m.) with free access to tap water and restricted access to certified rodent pellet. During the whole procedure, the regulations of the Hungarian Act No. XXVIII of 1998 on the protection and care of animals were strictly followed. Every procedure (handling, treatment, anaesthesia) executed in this experiment was approved by the Committee on Ethics of Animal Experiments of the University of Szeged and the Directorate of Food Safety and Animal Health Care, Government Agency of Csongrád County (Permit number: XXI./151/2013.). All efforts were made to minimize animal suffering.

### Administration of the antibiotic and green tea

The OECD Guidelines No. 417 [29] and No. 423 [30] were followed during the experiment. Dose selection was based on Ishizaki [31] and Woodnutt [32] and was adjusted to bioanalytical detection rate in the samples. The suspension of amoxicillin and clavulanic acid (7:1 w/w; dose: 100 mg amoxicillin/kg b.w.) was administered per os in single 1 ml/kg b.w. dose. The suspensions were prepared using either distilled water (AM group) or green tea infusion (AMG group). The suspensions were prepared freshly, no earlier than an hour before treatment, and were homogenized with vortex right before administration. Green tea infusion was prepared using 15 g of drug (green tea leaves) and 100 ml of boiling water, and after 5 min of maceration the extract was filtered on a tea-strainer. The room temperature filtrate was used for the preparation of the antibiotic suspension applied in the AMG group.

Access to rodent chow was restricted to approximately 10 g for a 16-h-period preceding treatment to follow the advice of the OECD Guideline No. 423. Treatment modelled human medication intake. Right before the treatment, the body weights of the animals were measured individually in order to precisely calculate the amount of suspension to be given. The oral administration of AM or AMG took place outside the cage, executed by a round-ended gastric tube. Six animals were treated daily in the mornings, consecutively, with 5-min delays.

### Blood sampling

The number of occasions for blood sampling were determined according to the pharmacokinetics of the test substance [32, 33]. Altogether, blood samples were taken six times: 30, 60, 90, 120, 180, and 240 min after the administration of AM/AMG. Considering the limited amount and occasions of blood sampling executable per animal (8 times at a maximum [34]), only 3 samples per animal were taken during the six-hour investigation time. To make sure that we obtain enough blood samples for the pharmacokinetic measurements, two animals were used for the total kinetic measurement. Accordingly, a total of twelve rats were used to obtain six data for each time point of the kinetics. The sampling protocol is given in Table 1.

**Table 1:** Sampling protocol. Times of blood sampling after treatment (30–240 min), showing which animals were used for blood sampling at each time point

	Code number of the animals											
	Amoxicillin (AM)						Amoxicillin + green tea (AMG)					
	1st treatment day			2nd treatment day			3rd treatment day			4th treatment day		
30′	4	5	6	10	11	12	16	17	18	22	23	24
60′	1	2	3	7	8	9	13	14	15	19	20	21
90′	4	5	6	10	11	12	16	17	18	22	23	24
120′	1	2	3	7	8	9	13	14	15	19	20	21
180′	4	5	6	10	11	12	16	17	18	22	23	24
240′	1	2	3	7	8	9	13	14	15	19	20	21

Before each blood sampling the animals had been warmed with an infrared lamp for thirty minutes in order to dilate the tail veins to ease blood sampling. At the first sampling occasion, the firmly but securely restrained rat was placed on a warm pad, and a Vasofix Certo 24 G ¾ (0,7 × 19 mm) intravenous catheter was placed in one of the lateral tail veins. To prevent the dislocation of the sampling needle a mild aether anaesthesia was provided under a ventilation hood to relax the animal when it was essential, by placing a small tube with a cotton bud containing 0.2 ml aether to the nostril of the animal for a couple of seconds with careful supervision. A minimum of 0.2 ml of blood samples were collected in a plastic tube, followed by injecting 0.12 ml of heparin into the catheter and finally closing and fixing it to the tail using an adhesive tape [34]. At each following sampling occasion, a couple of blood drops were let out of the catheter before taking blood. In-between the sampling occasions the rats were awoke. The need for anaesthesia during sampling was negligible. Administration and sampling volumes are shown in Table 2.

**Table 2:** Suspension (green tea +/− amoxicillin) volumes administered before sampling, and sampling volumes after treatment (30–240 min) (AM: amoxicillin, AMG: amoxicillin + green tea)

Group code	Animal code	Animal weight (g)	Administered suspension volume (ml)	Volumes of blood samples taken							
				30’	60’	90’	120’	180’	240’	300′	360′
AM	1	285	0.29	–	0.50	–	0.45	–	0.55	–	0.50
	2	292	0.29	–	0.50	–	0.40	–	0.60	–	0.60
	3	287	0.29	–	0.60	–	0.50	–	0.75	–	0.55
	4	285	0.29	0.48	–	0.45	–	0.50	–	0.80	–
	5	273	0.27	0.52	–	0.20	–	0.30	–	0.90	–
	6	281	0.28	0.45	–	0.40	–	0.55	–	0.60	–
	7	267	0.27	–	0.30	–	0.60	–	0.60	–	0.80
	8	285	0.29	–	0.65	–	0.70	–	0.70	–	0.90
	9	287	0.29	–	0.70	–	0.50	–	0.70	–	1.00
	10	285	0.29	0.70	–	0.60	–	0.50	–	0.25	–
	11	288	0.29	0.70	–	0.30	–	0.30	–	0.20	–
	12	290	0.29	0.65	–	0.60	–	0.55	–	0.30	–
AMG	13	296	0.30	–	0.70	–	0.70	–	0.70	–	0.70
	14	302	0.30	–	0.60	–	0.60	–	0.70	–	0.50
	15	294	0.29	–	0.45	–	0.70	–	0.60	–	0.60
	16	298	0.30	0.70	–	0.55	–	0.70	–	0.60	–
	17	301	0.30	0.70	–	0.30	–	0.50	–	0.70	–
	18	287	0.29	0.60	–	0.60	–	0.55	–	0.50	–
	19	311	0.31	–	0.35	–	0.50	–	0.45	–	0.50
	20	306	0.31	–	0.65	–	0.65	–	0.70	–	0.90
	21	312	0.31	–	0.75	–	0.60	–	0.50		0.60
	22	307	0.31	0.50	–	0.50	–	0.7	–	0.70	–
	23	292	0.29	0.50	–	0.80	–	1.00	–	1.50	–
	24	301	0.30	0.45	–	0.30	–	0.60	–	0.70	–

After the sampling period the animals were over-anaesthetized (euthanized) with isoflurane inhalation. A precision vaporizer with induction chamber and waste gas scavenger was used. An isoflurane concentration of over 5% in 100% oxygen was slowly achieved and continued until one minute after breathing stopped.

### Quantification of amoxicillin in plasma samples

During blood sample processing the serum was separated from the clotted fraction by centrifugation (2700 G, 10 min). 50 μl of each centrifuged plasma sample was pipetted into 250 μl MeOH on a 0.5 mL 96-well Costar plate and cooled to 4 °C for 1 h. Samples were centrifuged (2000 rcf) for 30 min at 8 °C. 50 μl supernatant was taken and evaporated. Calibration standards were prepared and injected directly into the LC-MS/MS apparatus. The concentration of amoxicillin and clavulanic acid was measured using an Agilent 1290 HPLC coupled to an Agilent 6470A Triple Quadrupole Mass Spectrometer equipped with an RP column (Luna 3 μ C8(2) 100 Å 50 × 2 mm; Phenomenex 00B-4251-B0). Isocratic elution was applied (85% of 0.1% HCOOH aqueous solution; 15% of 0.1% HCOOH in acetonitrile), the flow rate was 0.5 ml/min and column temperature was set to 40 °C.

### Statistical analysis

Descriptive and summary statistics were calculated. All results are presented as means ± standard error of the mean (SEM). The area under the amoxicillin plasma concentration-time curves (AUC_0–240_) were calculated. The plasma concentrations and AUC curves for amoxicillin in the AM and AMG groups were compared using paired Student’s t-test. A *p* < 0.05 was considered as significant. All calculations were performed with the GraphPad software (GraphPad Prism v. 8.0.1).

## Results

**Fig. 1:**
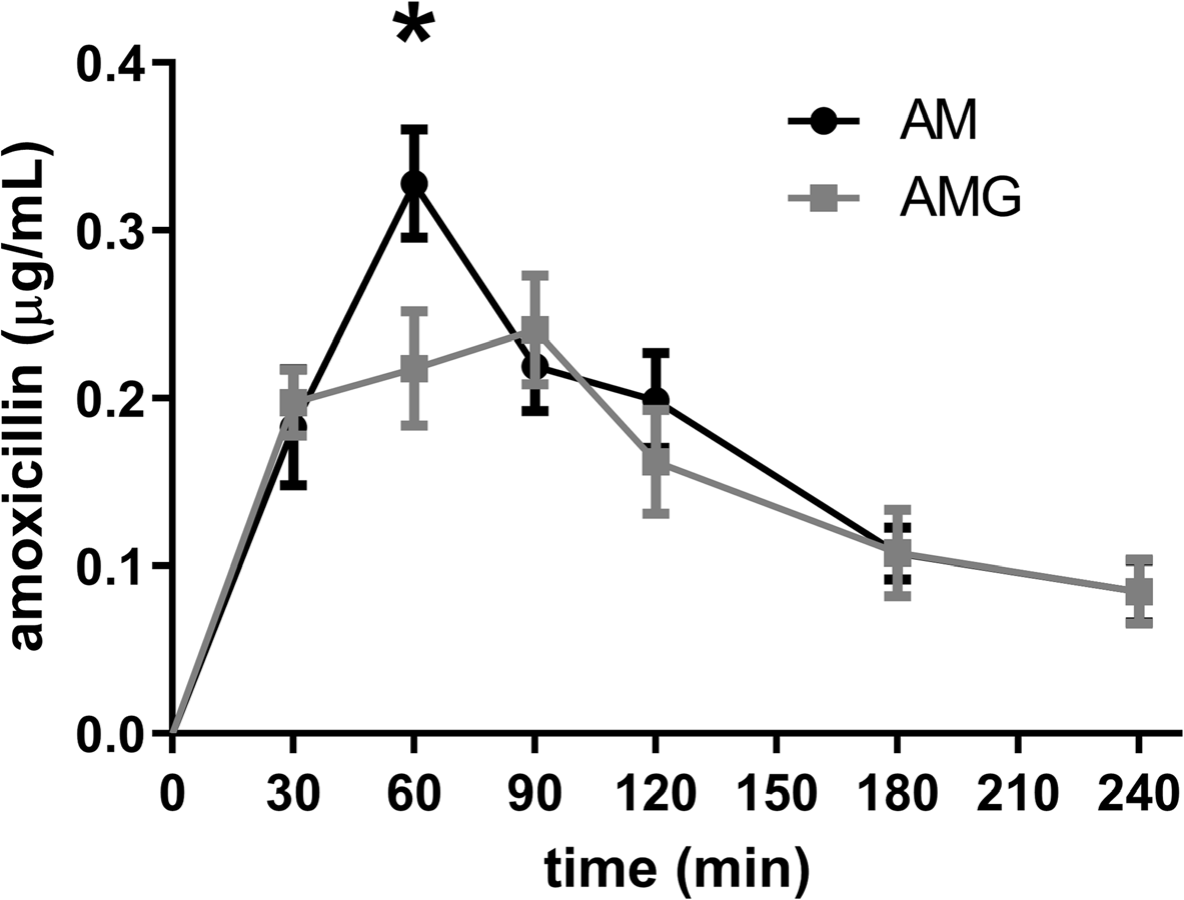
Change in the plasma concentration of amoxicillin in the AM and AMG groups. Data are shown as mean ± SEM (*p < 0.05)

Total polyphenol content of green tea was 12.31 ± 0.41% PGE. The plasma concentration of amoxicillin was monitored by LC-MS/MS measurements in 30–60-min-intervals for 240 min. Changes of amoxicillin level are shown in Fig. 1. A significant between-group difference in the plasma concentrations of amoxicillin was observed at 60 min only, while at other time points the difference between the groups did not reach the level of statistical significance. However, the highest plasma level of amoxicillin was significantly lower in the AMG group, which suggests an interaction between green tea polyphenols and the antibiotic.

**Fig. 2:**
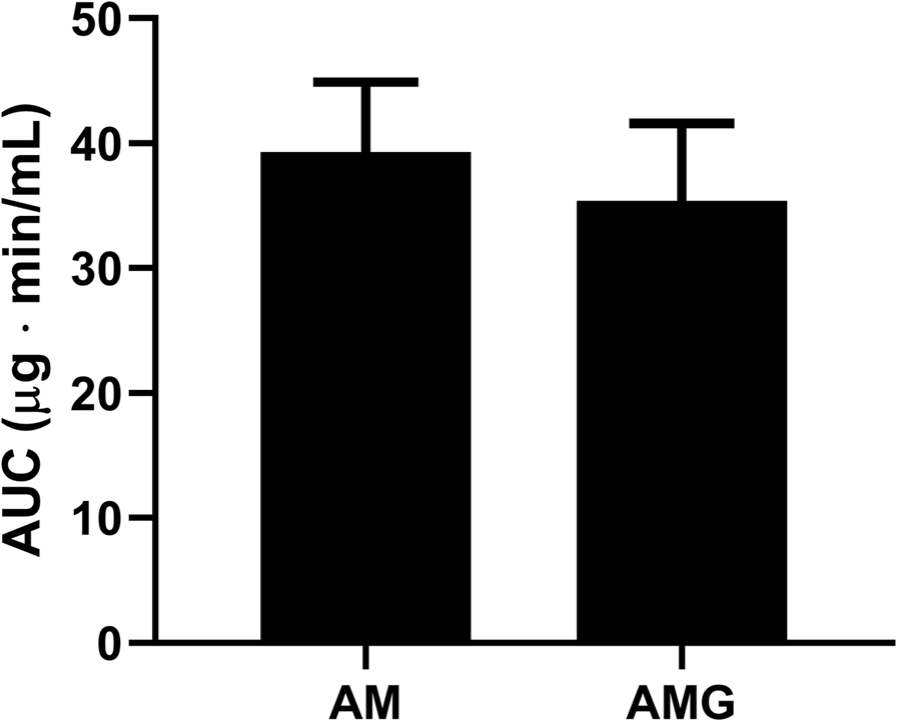
AUC values in the AM and AMG groups. Data are shown as mean ± SEM

Comparing the AUC_0–240_ for the two treatment groups (AM and AMG, *N* = 6 both) a slight decrease in amoxicillin absorption was observed in the AMG group, however, the difference did not reach statistical significance (Fig. 2).

## Discussion

The aim of our study was to assess the presumable interaction between a frequently used antibiotic (the combination of amoxicillin and clavulanic acid) and green tea infusion, in a setting mimicking the intake of the medicine with green tea.

The investigated tea was analytically characterized and was proved to be rich in polyphenols [35]. Based on literature, in vitro experiments demonstrate that catechins, the main polyphenolic compounds of green tea, do influence the antimicrobial activity of amoxicillin, and the in vitro antibacterial effect of amoxicillin against *Helicobacter pylori* is significantly enhanced by the presence of epigallocatechin gallate [36]. Similarly, the synergistic effect of catechins and amoxicillin against *Escherichia coli* is also reported [17]. However, these in vitro findings have no relevance for the clinical efficacy of amoxicillin, since polyphenols have very low bioavailability, thus, in fact, the gastrointestinal tract is the space of potential interactions between these compounds and amoxicillin. An opposite effect was also observed in mice infected with methicillin-resistant *Staphylococcus aureus*: in an in vivo experiment green tea extract was found to weaken the effect of amoxicillin after enteral administration (gastric perfusion) [37]. Our results confirm this finding, and offer a possible explanation for green tea ameliorating the antibacterial efficacy of amoxicillin treatment.

Not only green tea infusion, but also concentrated food supplements may contain high doses of catechins (up to 1000 mg [38])). As these supplements are likely to be taken concomitantly with medications, the occurrence of an interaction between polyphenols/catechins and amoxicillin has relevance in real-life settings.

Although the AUC of amoxicillin did not decrease significantly during the observation period in our in vivo experiment, the concomitant administration of green tea induced a significant reduction in the peak plasma concentration of the antibiotic [37]. Despite the limitation of the study (small but reasonable sample size), our findings support the findings of a previously published animal experiment, and draw attention to the possibility of a pharmacokinetic interaction between green tea and amoxicillin upon human application. Further studies are needed to explore this interaction regarding per os human therapeutic use.

## Conclusions

Due to its high polyphenol content green tea may interact with the absorption of amine-containing molecules, including antibiotics. This is the first report on the in vivo assessment of the interaction between a widely used antibiotic, amoxicillin, and green tea. Although the concomitant consumption of green tea did not alter the AUC of the antibiotic significantly within the observation period, the decrease in the peak plasma concentration of amoxicillin refers to an interaction of potential clinical relevance, worth being studied in human settings.

## Data Availability

The datasets used and/or analysed during the current study are available from the corresponding author on reasonable request.

## Abbreviations

AM: Amoxicillin
AMG: Amoxicillin plus green tea
AUC: Area under the curve
AUC_0–240_: Area under curve in the first 240 min
EGCG: Epigallocatechin gallate
HCOOH: Formic acid
HPLC: High performance liquid chromatography
LC-MS/MS: Liquid chromatography/mass spectrometry
LC-MS/MS: Liquid chromatography/tandem mass spectrometry
MeOH: Methanol
MRSA: Methicillin-resistant *Staphylococcus aureus*
OECD: Organisation for Economic Co-operation and Development
PGE: Pyrogallol equivalent
rcf: relative centrifugal force
SEM: Standard error of mean
WHO: World Health Organization

## References

[1] WHO. World antibiotic awareness week: WHO; 2018.

[2] Bushra R, Aslam N, Khan AY (2011). Food-drug interactions. Oman Med J.

[3] Leibovitch ER, Deamer RL, Sanderson LA (2004). Food-drug interactions: careful drug selection and patient counseling can reduce the risk in older patients. Geriatrics.

[4] Adamczyk Bartosz, Simon Judy, Kitunen Veikko, Adamczyk Sylwia, Smolander Aino (2017). Tannins and Their Complex Interaction with Different Organic Nitrogen Compounds and Enzymes: Old Paradigms versus Recent Advances. ChemistryOpen.

[5] (2018) Tea in 2018: annual market overview. https://www.euromonitor.com/tea-in-2018-annual-market-overview/report. Accessed 3 Jun 2019.

[6] (2018) Current market situation and medium term outlook. Hangzhou.

[7] Graham HNN (1992). Green tea composition, consumption, and polyphenol chemistry. Prev Med (Baltim).

[8] Zhang Jia-Yi, Liao Yu-Huang, Lin Ying, Liu Qiang, Xie Xiao-Ming, Tang Lu-Ying, Ren Ze-Fang (2019). Effects of tea consumption and the interactions with lipids on breast cancer survival. Breast Cancer Research and Treatment.

[9] Mineharu Y., Koizumi A., Wada Y., Iso H., Watanabe Y., Date C., Yamamoto A., Kikuchi S., Inaba Y., Toyoshima H., Kondo T., Tamakoshi A. (2009). Coffee, green tea, black tea and oolong tea consumption and risk of mortality from cardiovascular disease in Japanese men and women. Journal of Epidemiology & Community Health.

[10] Centre for the Promotion of Imports, CBI Ministry of Foreign Affairs. https://www.cbi.eu/market-information/tea/trade-statistics. Accessed 3 Jun 2019.

[11] Anand J, Rai N, Kumar N, Gautam P. Green tea: a magical herb with miraculous outcomes. Int Res J Pharm. 2012;3:139–48.

[12] Serafini M, Del Rio D, Yao DN, et al. Health benefits of tea. In: Benzie IFF, Wachtel-Galor S, editors. Herbal medicine: biomolecular and clinical aspects. 2nd ed: CRC Press/Taylor & Francis; 2011. 22593937

[13] Albassam A, Markowitz J (2017). An appraisal of drug-drug interactions with green tea (Camellia sinensis). Planta Med.

[14] Reygaert WC. The antimicrobial possibilities of green tea. Front Microbiol. 2014;5(434) 10.3389/fmicb.2014.00434. 10.3389/fmicb.2014.00434 PMC4138486 25191312

[15] Hacioglu M, Dosler S, Birteksoz Tan AS, Otuk G (2017). Antimicrobial activities of widely consumed herbal teas, alone or in combination with antibiotics: an in vitro study. PeerJ.

[16] (2008) Green tea helps beat superbugs, study suggests. ScienceDaily.

[17] Noormandi A, Dabaghzadeh F (2015). Effects of green tea on Escherichia coli as a uropathogen. J Tradit Complement Med.

[18] Gillies Malcolm, Ranakusuma Anggi, Hoffmann Tammy, Thorning Sarah, McGuire Treasure, Glasziou Paul, Del Mar Christopher (2014). Common harms from amoxicillin: a systematic review and meta-analysis of randomized placebo-controlled trials for any indication. Canadian Medical Association Journal.

[19] Babraczy B, Benkő R, Borbás I, et al. Policy brief. Promoting the appropriate use of antibiotics to contain antibiotic resistance in human medicine in Hungary. Copenhagens; 2018.

[20] Lutz Mariane, Espinoza Julio, Arancibia Aquiles, Araya Magdalena, Pacheco Isoida, Brunser Oscar (1987). Effect of structured dietary fiber on bioavailability of amoxicillin. Clinical Pharmacology and Therapeutics.

[21] Neu HC (1974). Antimicrobial activity and human pharmacology of amoxicillin. J Infect Dis.

[22] Neuvonen PJ, Elonen E, Pentikäinen PJ (1977). Comparative effect of food on absorption of ampicillin and pivampicillin. J Int Med Res.

[23] Eshelman FN, Spyker DA (1978). Pharmacokinetics of amoxicillin and ampicillin: crossover study of the effect of food. Antimicrob Agents Chemother.

[24] Welling P.G., Huang H., Koch P.A., Craig W.A., Madsen P.O. (1977). Bioavailability of Ampicillin and Amoxicillin in Fasted and Nonfasted Subjects. Journal of Pharmaceutical Sciences.

[25] Ginsburg CM, McCracken GH, Thomas ML, Clahsen J. Comparative pharmacokinetics of amoxicillin and ampicillin in infants and children. Pediatrics. 1979;64:627–31. 492836

[26] Schmidt Lars E., Dalhoff Kim (2002). Food-Drug Interactions. Drugs.

[27] Passat DN (2012). Interactions of black and green tea water extracts with antibiotics activity in local urinary isolated Escherichia coli. J Al-Nahrain Univ Sci.

[28] EDQM (2005). Determination of tannins in herbal drugs. European Pharmacopoeia 5.0.

[29] OECD. Test No, vol. 417: Toxicokinetics; 2010.

[30] OECD (2002). Test no. 423: acute Oral toxicity - acute toxic class method.

[31] Ishizaki Junko, Tsuda Tomoko, Suga Yukio, Ito Satsuki, Arai Kunizo, Sai Yoshimichi, Miyamoto Ken-ichi (2012). Change in Pharmacokinetics of Mycophenolic Acid as a Function of Age in Rats and Effect of Coadministered Amoxicillin/Clavulanate. Biological and Pharmaceutical Bulletin.

[32] Woodnutt Gary, Berry Valerie (1999). Efficacy of High-Dose Amoxicillin-Clavulanate against Experimental Respiratory Tract Infections Caused by Strains ofStreptococcus pneumoniae. Antimicrobial Agents and Chemotherapy.

[33] (2018) CLAVULIN Product Monograph.

[34] NC3Rs National Centre for the replacement refinement and reduction of animals in research, Temporary cannula. PMC3284057 22368436

[35] Dalluge JJ, Nelson BC (2000). Determination of tea catechins. J Chromatogr A.

[36] Yanagawa Y, Yamamoto Y, Hara Y, Shimamura T (2003). A combination effect of epigallocatechin gallate, a major compound of green tea catechins, with antibiotics on *Helicobacter pylori* growth in vitro. Curr Microbiol.

[37] Peng Qing, Huang Yuanchun, Hou Bing, Hua Dexing, Yao Fen, Qian Yuanshu (2010). Green tea extract weakens the antibacterial effect of amoxicillin in methicillin-resistantStaphylococcus aureusinfected mice. Phytotherapy Research.

[38] Younes M, Aggett P, Aguilar F, et al. Scientific opinion on the safety of green tea catechins. EFSA J. 2018;16(5239) 10.2903/j.efsa.2018.5239. 10.2903/j.efsa.2018.5239 PMC7009618 32625874

